# Biofriendly micro/nanomotors operating on biocatalysis: from natural to biological environments

**DOI:** 10.1007/s41048-020-00119-6

**Published:** 2020-10-31

**Authors:** Ziyi Guo, Jian Liu, Da-Wei Wang, Jiangtao Xu, Kang Liang

**Affiliations:** 1 School of Chemical Engineering, University of New South Wales, Sydney, NSW 2052, Australia; 2 Australian Centre for NanoMedicine, University of New South Wales, Sydney, NSW 2052, Australia; 3 Graduate School of Biomedical Engineering, University of New South Wales, Sydney, NSW 2052, Australia

**Keywords:** Micro/nanomotors (MNMs), Biofriendly MNM design, Biocatalysis

## Abstract

Micro/nanomotors (MNMs) are tiny motorized objects that can autonomously navigate in complex fluidic environments under the influence of an appropriate source of energy. Internal energy driven MNMs are composed of certain reactive materials that are capable of converting chemical energy from the surroundings into kinetic energy. Recent advances in smart nanomaterials design and processing have endowed the internal energy driven MNMs with different geometrical designs and various mechanisms of locomotion, with remarkable travelling speed in diverse environments ranging from environmental water to complex body fluids. Among the different design principals, MNM systems that operate from biocatalysis possess biofriendly components, efficient energy conversion, and mild working condition, exhibiting a potential of stepping out of the proof-of-concept phase for addressing many real-life environmental and biotechnological challenges. The biofriendliness of MNMs should not only be considered for *in vivo* drug delivery but also for environmental remediation and chemical sensing that only environmentally friendly intermediates and degraded products are generated. This review aims to provide an overview of the recent advances in biofriendly MNM design using biocatalysis as the predominant driving force, towards practical applications in biotechnology and environmental technology.

## INTRODUCTION

Inspired by the diverse molecular motors in nature (Cross 1997; Thomas and Thornhill 1998), recent rapid explosion of materials research and nanotechnology allowed us to explore the potential of artificial micro/nanomotor (MNM) systems for numerous applications (Fernández‐Medina *et al*. 2020). MNM systems present unique properties including controllable motion (Arque *et al*. 2019), high cargo loading efficiency (Ma *et al*. 2017), strong towing force (Sanchez *et al*. 2010) and ease of surface functionalization (Restrepo-Perez *et al*. 2014). Accordingly, multifarious demonstrations and applications have been developed by exploiting their material tunability (Jurado-Sánchez *et al*. 2017; Ning *et al*. 2018; Wang *et al*. 2016), sensing capabilities (Jurado-Sanchez 2018; Jurado-Sánchez and Escarpa 2017) and controllability (Eskandarloo *et al*. 2017) for cargo transportation (Ma *et al*. 2015), environmental remediation (Jurado-Sánchez and Wang 2018; Vilela *et al*. 2016; Ying *et al*. 2019) and drug delivery (Guo *et al*. 2019). A significant amount of MNMs reported to date have focused on the motion manipulation mechanism with potentially environmentally hazardous metal-based catalysts regardless of the manufacturing cost or the naturally feasible working conditions, posing difficulties in the real-world applications of MNMs both *in vivo* and in the natural environment. Accordingly, biocatalysts in general possess higher catalytic efficiency in mild working conditions, endowing biocatalytic MNMs with low cost and high energy efficiency. The great biocompatibility of the MNMs systems is expected to play an essential role in the development of stepping out of the proof-of-concept phase for addressing many real-life environmental and biotechnological challenges.

Recent advances in biocompatible MNMs research have made them become promising candidates for addressing many bio-related challenges owing to their characteristics. These small MNMs present an advantage in overcoming cellular barriers and improving cellular uptake, which made them good nanocarrier substitutes for drug delivery (Esteban-Fernández de Ávila *et al*. 2018a, b; Gao *et al*. 2019; Guo *et al*. 2019; Wang *et al*. 2019b). However, not only in nanomedicine, employing MNMs in many other areas, for example, cargo delivery (Sanchez *et al*. 2010), chemical sensing (Orozco *et al*. 2013), polluted water treatment (Orozco *et al*. 2014) and reaction catalysis, requires the MNMs to be biofriendly by generating environmental benign intermediates and degraded products. Although the recent development of MNMs has been extensively documented in several reviews, with the majority being focused on the composition, motion mechanism or applications (Sun *et al*. 2019), here we provide an overarching perspective on MNMs utilizing biocatalysis as the driving force, with a special focus on their biocompatibility in both biological system and the natural environment. Utilizing biocatalysis as the driving force for MNMs offers unique advantages, apart from the excellent specificity and catalytic efficiency, the inherent biocompatible nature of enzymes allows MNMs to operate in mild, biofriendly environments by generating little or no toxic chemical wastes, placing them as an ideal candidate for addressing unmet challenges in biomedicine and the environment.

**Figure 1:**
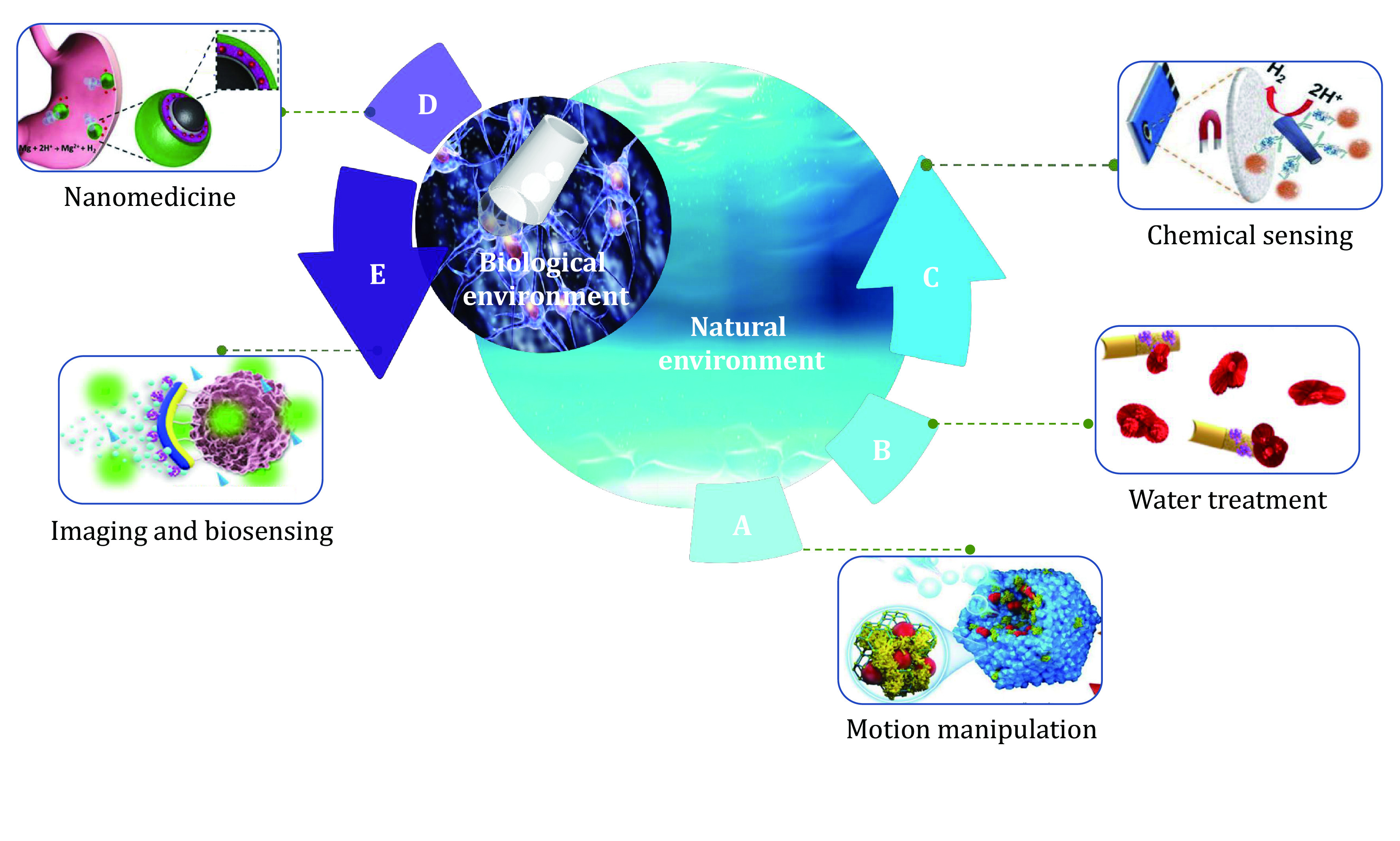
Overview of biofriendly MNMs driven by biocatalysis for environmental and biotechnological applications. Reproduced with permission: **A** WILEY-VCH (Gao *et al*. 2019); **B** American Chemical Society (Kiristi *et al*. 2015); **C** WILEY-VCH (Mayorga‐Martinez and Pumera 2019); **D** Springer Nature (de Ávila *et al*. 2017); **E** Elsevier (Zhao *et al*. 2020)

In this review, we select the most recent and representative work in this field, from our own work and the work by others, to showcase this rapid emerging field. In the first section, we orient our focus on the recent advances in biofriendly MNM design using biocatalysis as the predominant driving force, including choices of biocatalysts and MNM building materials, morphological and size control, and strategies for biocatalysts incorporation. Next, we highlight the practical applications in biomedical and the environmental technology enabled by biocatalytic MNMs, including motion manipulation, water treatment, chemical sensing, nanomedicine, bioimaging and biosensing (Fig. 1). In the last section, we provide an overview and outlook of the possible biocompatible designs that could be adapted for future use.

## BIOCOMPATIBLE AND BIOCATALYTIC MNMS DESIGN

### Biocatalytic reactions as the driving force

In general, MNM propulsion mechanisms can be categorized into physical driven propulsion (*e.g*. interfacial surface tension), chemical fuel consumption (*e.g*. H_2_O_2_) and external field propulsion such as electrical field, magnetic field, light and ultrasound (Tu *et al*. 2017). The propulsion strategies in MNM design exert significant influence on their systematic biocompatibility and consequently, applications. Biocatalytic reactions using enzymes as the catalysts generally exhibit higher energy utilization efficiency and superior biocompatibility in biofriendly environment owing to their excellent substrate selectivity and product turnover rate in mild operating conditions. Accordingly, enzyme-powered MNMs have been extensively explored, where the enzymatic reaction for gas generation being the most dominant focus (Chen *et al*. 2019; Ma *et al*. 2016). Biocatalytic MNMs can be propelled by bubble buoyancy (Kumar *et al*. 2018), the thrust from the bubble ejection (Gao *et al*. 2012), and the detachment force from the released bubbles (Wang *et al*. 2006). Maria and co-workers reported an asymmetric mesoporous silica nanomotor functionalized with single-stranded DNA and catalase (Simmchen *et al*. 2012). The immobilized enzymes produced oxygen bubbles as the driving force from catalytic decomposition of hydrogen peroxide. With functionalized DNA strand on their surface, the nanomotors were capable of capturing and transporting cargos with noncomplementary single-stranded DNA molecule.

Currently, the types of the biochemical fuels for enzyme-powered MNMs are mostly restricted to H_2_O_2_, urea, glucose, and hydrazine (Gao *et al*. 2014) operating from one or a combination of enzymes (*e.g*. catalase, glucose oxidase, and urease). Based on the bio-specificity of the chemical fuels, some of the enzyme-powered MNMs can be applied for chemotaxis-driven targeting transportation (Joseph *et al*. 2017). Battaglia and coworkers reported an enzyme-propelled nanoscopic swimmer by encapsulating glucose oxidase in polymersomes (Joseph *et al*. 2017). The nano-swimmer exhibited chemotaxis behavior in response to external glucose concentration gradient, which led to the motion towards higher glucose regions. With the aid of low-density lipoprotein receptor-related protein 1 (LRP-1) functionalization, the chemotactic behavior of the nanoswimmers demonstrated improved ability in crossing the blood–brain barrier. In another work, different enzymes, including catalase, urease and ATPase, were coated on the liposomes to fabricate biocatalytic motors with positive chemotaxis, negative chemotaxis, and tunable chemotaxis, respectively (Somasundar *et al*. 2019). The different motion behaviors of the motors were believed to be originated from positive chemotaxis and solute –phospholipid-based negative chemotaxis, as results of different enzyme catalysis routes.

Hitherto, the most popular biofriendly biocatalytic MNMs are propelled by limited types of fuels, which are far less than the traditional catalytic reactions reported for motor propulsion using more toxic inorganic catalysts. Due to the restriction of biocatalytic reaction conditions such as limited pH and temperature ranges, the utilization of other catalytic reactions to biofriendly systems remained a challenge. To minimize the use of materials hazardous to human health and the environment, the exploitation of more diverse biocatalytic reactions in biological system is expected to be applied as alternatives for biocatalytic MNM design in the future.

### Material choices for better biocompatibility

A variety of materials, including inorganic materials (Coopersmith 2017), organic materials (Wang and Pumera 2017) and hybrid materials (Khezri and Pumera 2019), with diverse chemical compositions have been employed as biocatalytic MNMs matrix. For environmental applications such as water remediation, the toxicity and stability of the material itself are dominant factors for consideration. For biological applications, inappropriate choice of materials could result in inflammation, immunoreaction or even lethal disease from undesired material–biological interaction (Wang *et al*. 2019b).

Inorganic materials, including mesoporous silica (SiO_2_) (Llopis-Lorente *et al*. 2019; Ma and Sánchez 2017), graphene oxide (GO) (Yu *et al*. 2017) and titanium dioxide (TiO_2_) (Gáspár 2014), are among the most employed materials to construct biofriendly MNMs due to their facile synthetic procedure, adequate stability and ease of surface modification. It is a general consensus that these inorganic materials are abundant in nature with negligible toxicity towards the surrounding environment and living systems at appropriate dosages (Wang *et al*. 2014).

Besides diverse inorganic materials, synthetic polymers have also been developed to fabricate biofriendly MNMs due to their excellent molecular tunability and facile synthetic procedures, which can equip polymer-based MNMs with proper physicochemical properties, flexible morphologies, diverse functionalities and non-toxicity (Wong *et al*. 2016). The diversity of biofriendly polymers imparts significant convenience for the construction of multifunctional MNMs in various aspects from natural environment to biological environment (Somasundar *et al*. 2019; Wu *et al*. 2014). For example, Wu and the coworkers reported a polyelectrolyte based Janus micromotor system for NIR-light-responsive drug delivery with half coated gold layer (Wu *et al*. 2014). Biocatalytic enzyme catalase was immobilized on the gold layer to propel the motors using hydrogen peroxide as the fuel in the surrounding environment. The enzymatic motor system exhibited higher catalytic efficiency compared to inorganic Pt-based synthetic motors.

To further extend the compatibility and responsiveness of MNMs in natural and biological environments, hybrid materials that possess desired materials properties from inorganic, organic and even biological components, such as facile synthetic process, the ease of surface functionalization, great porosity and biocompatibility, have been exploited (Khezri and Pumera 2019). Biological components, such as proteins, cell membranes, and even whole cells can also be employed in the fabrication process for MNM surface functionalization and propulsion (Esteban-Fernandez de Avila *et al*. 2018). Employing hybrid materials in MNM design have tremendous advantages due to their great flexibility and biocompatibility to the surrounding, and they are relatively more sensitive to the specific working environment (Guo *et al*. 2019). With well-developed fabrication techniques of artificial materials, nano-biohybrid MNMs with greater biocompatibility are expected to be the rising star in MNMs design in future studies.

### Morphological and size control

Recent advances in MNMs research have endowed them various sophisticated shapes and size with great flexibility, which have shown significant impact on their motion patterns and energy conversion efficiency (Patino *et al*. 2018). With different geometric distribution, the MNMs exhibit structure-dependent motion behavior with various resulting speed and propulsive force. Sánchez and coworkers studied the influence of enzyme distribution and quantity on the motion behavior with urease-conjugated polystyrene (PS) and silicon dioxide-coated polystyrene (PS@SiO_2_) micromotors (Patino *et al*. 2018). Their results demonstrated that the amount of conjugated enzymes was found to be nonlinear to the motion speed and propulsive force resulted from the enzymatic reaction-induced propulsion.

To address biomedical challenges with the aid of MNMs, the size of MNM is one of the most important factors that ultimately determines whether the MNM can effectively penetrate the cell membrane to deliver the loaded therapeutics. It is widely accepted that nanoparticles with diameters ranging from 20 to 200 nm have advantages in overcoming cellular barriers for drug delivery (Sun *et al*. 2019). However, most of the existing MNMs systems have a size range at the microscale, which limits their potential for *in vivo* delivery applications. To circumvent this issue, Wilson and coworkers found that the addition of poly(ethylene glycol) (PEG) was conducive to size and shape control in nanoscale, allowing small polymersomes to transform into stomatocytes (Sun *et al*. 2019). This ultra-small stomatocyte nanomotor has a diameter around 100 nm with the capability of encapsulating catalase enzyme as the engine that converts H_2_O_2_ into oxygen bubble to propel the nanomotors. The ability to encapsulate enzymes in the inner compartment of this stomatocyte made it promising nanovesicle candidate for protein delivery and bioimaging. With the small size range and rapid motion, this nanomotor exhibited excellent cell uptake efficiency.

### Strategies for biocatalysts incorporation

Due to the great catalytic efficiency, high substrate specificity and selectivity, enzymes are widely applied as natural catalysts in various industrial chemical and pharmaceutical production processes. However, being limited by the intrinsic low stability and flexibility, free enzymes showed less environmental tolerance to extreme temperature and pH (Liang *et al*. 2015). To enhance the stability, recyclability and catalytic efficiency of enzymes, many studies have looked into immobilizing enzymes onto various surfaces which exhibited distinct stability improvement without loss of catalytic efficiency (Zhao *et al*. 2018). The immobilized enzymes on nanoscale support exhibited significantly enhanced mass transfer efficiency and higher diffusion efficiency. For biocatalytic MNMs, strategies for immobilizing enzymes to the motor framework generally include adsorption, encapsulation and covalent attachment (Wong *et al*. 2019). Ma and coworkers reported a dual-enzyme-functionalized self-propelled therapeutic nanosystem for synergetic photodynamic therapy (PDT) and starvation therapy (ST) (You *et al*.^2019^). The core NaYF4:Yb,Tm@NaYF4 nanoparticles (UCNPs) and 5,10,15,20-tetrakis(4aminophenyl)porphyrin (TAPP) were encapsulated in ZIF-8 metal-organic framework (MOF) particles through one-pot synthesis, forming UCNPs/TAPP@ZIF-8 (UTZ). To covalently graft the catalase and glucose oxidase enzymes, the UTZ particles were first modified with glutaraldehyde (GA) and then incubated with the enzymes in PBS for 16 h. The Yb^3+^ in the catalase/GOx functionalized UTZ is capable of harvesting energy from 980 nm NIR light and transfer to TAPP for ^3^O_2_–^1^O_2_ transformation. The cascade reaction from dual enzymes propelled the motors in solution containing glucose, which greatly enhanced the diffusivity of the micromotors and the cellular uptake efficiency.

In another work, Sánchez and coworkers reported a mesoporous silica nanomotor system with pH-responsive supramolecular nanovalves (Llopis-Lorente *et al*. 2019). The silica nanomotors were surface functionalized with benzimidazole group and capped with cyclodextrin-modified urease to prevent the inner cargo from leaking. The urease acted as the biocatalytic engines to propel the motors and the grafted benzimidazole group acted as the valves with the formation of inclusion complexes to release cargo in acidic environments.

Besides covalent attachment, encapsulation is also popular for enzyme immobilization with high loading efficiency. Wilson and coworkers reported an enzyme-powered polymeric stomatocytes nanomotor with catalase and glucose oxidase. The stomatocytes were first formed with block copolymer poly(ethylene glycol)_44_-*b*-poly(styrene)_167_ and the enzymes were mixed with the glassy stomatocytes followed by closing of the stomatocyte neck with solvent addition method. The constructed motors were propelled by the enzymatic reaction and exhibited high speed in biologically relevant fuels. Our group reported a universal and facile strategy to encapsulate almost any kinds of biocatalysts in MOF nanoparticles through a one-step biomineralization process (Liang *et al*. 2015). Our results demonstrated that enzymes, DNA, proteins, polysaccharides, and even living cells can be encapsulated in MOF particles with high loading efficiency (Liang *et* *al*. 2016a, b; 2017) showing great promise in advanced hybrid MNM design for diverse applications.

## APPLICATIONS IN BIOMEDICAL AND ENVIRONMENTAL TECHNOLOGY

### Motion manipulation

Due to the chemical-to-kinetic energy conversion ability, the great mobility against diffusion limit, and continuous stirring of the surrounding environment, MNMs hold great promise for diverse applications from environmental remediation to nanomedicine. Many fundamental studies on MNMs systems are primarily focused on motion and speed manipulation but lack of actual practical applications. However, these fundamental studies shed light to future development in more practical applications employing MNMs. Here we provide a non-comprehensive highlight of several studies on the enzyme-powered, biofriendly MNMs for motion manipulation towards future practical applications.

**Figure 2:**
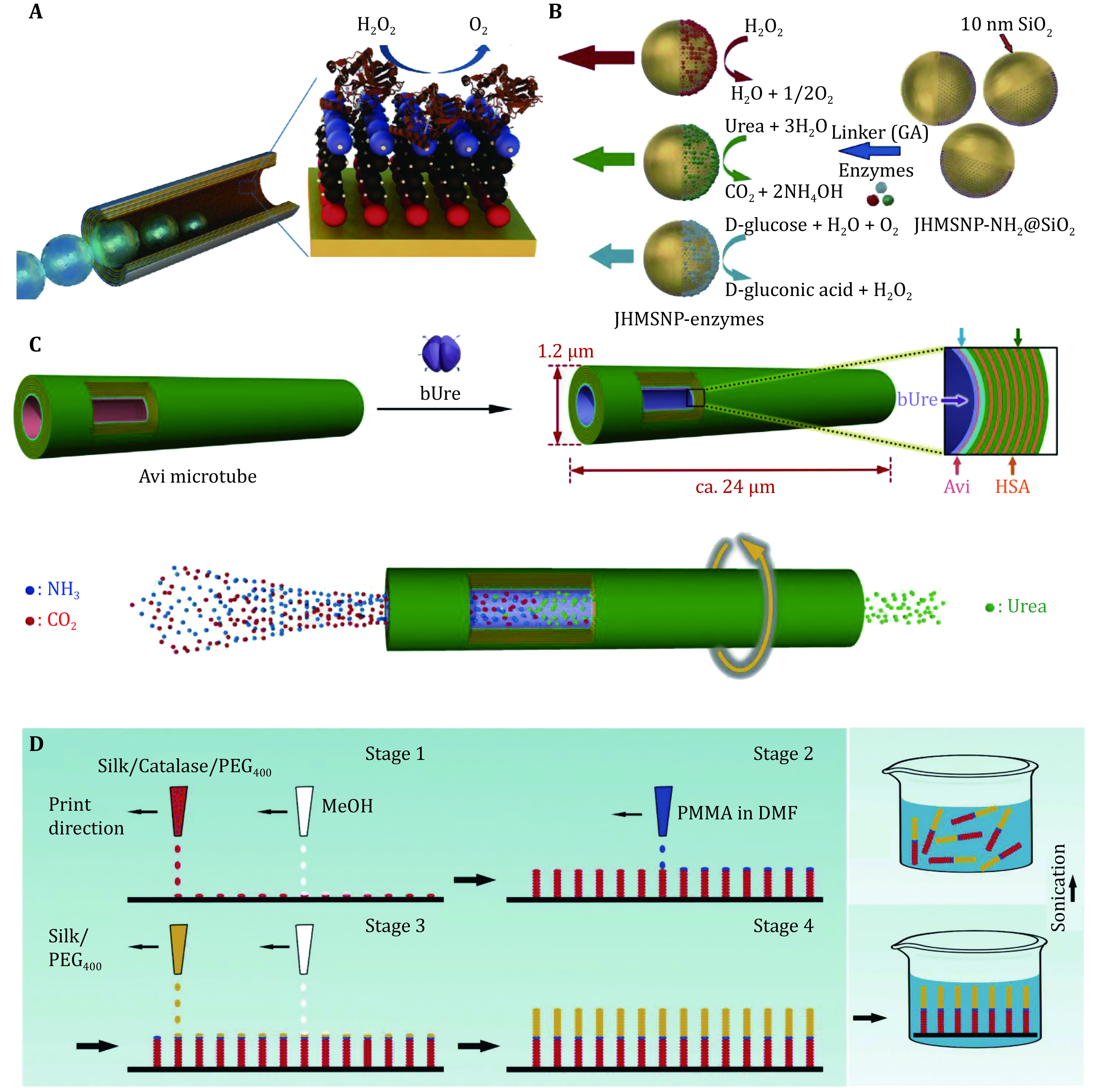
Micromotor systems for motion control and cargo delivery. **A** Open view of the hybrid biocatalytic micro-engine with surface modification of inner Au layer and enzymatic decomposition of peroxide fuel. Reproduced with permission of American Chemical Society (Sanchez *et al*. 2010). **B** Schematic illustration of enzymatic hollow mesoporous silica Janus nanomotors. Reproduced with permission of Springer Nature (Kumar *et al*. 2018). **C** Schematic illustration of preparation of avidin/ biotinylated urease (Avi/bUre) microtube and swimming Avi/bUre microtube with non-bubble propulsion and self-rotation. Reproduced with permission of Wiley‐VCH (Sugai *et al*. 2019). **D** Schematic of the reactive inkjet printing process for manufacturing biocatalytic micro-rockets. Reproduced with permission of WILEY‐VCH (Gregory *et al*. 2016)

The primary design of biocompatible micromotors is simply composed of a micro-engine body and a propelling module. Schmidt and coworkers reported a rolled-up Ti/Au microtube with covalently bound catalase on the inner layer (Fig. 2A) (Sanchez *et al*. 2010). The Au layer was functionalized with self-assembled monolayers (SAMs) of 3-mercaptopropionic acid (3-MPA) coupled with 1-ethyl-3[3-dimethylaminopropyl] carbodiimide hydrochloride (EDC) and N-hydroxylsulfosuccinimide (Sulfo-NHS) to form covalent bond between catalase and the inner layer. Catalase allowed oxygen bubble generation from the chemical fuel hydrogen peroxide. As a result, the continuous micromotor motion was achieved by bubble propulsion, which allowed a significant higher carrying force of over 16.44 pN as compared to Pt-based micro-engines.

Multi-enzyme cascade reactions can also be employed in biocompatible enzyme-powered nanomotors. In the study by Sanchez and coworkers, silica nanoparticle template was coated with a mesoporous silica shell and etched with Na_2_CO_3_ to form hollow mesoporous silica nanoparticles (HMSNPs), which were further modified with amino groups and half-capped with a 10 nm silicon dioxide layer (Fig. 2B) (Ma *et al*. 2015). The enzymes, including catalase, urease and glucose oxidase were covalently conjugated to the uncapped side of the HMSNPs to propel the nanomotors with asymmetric biocatalytic reactions using biologically benign fuels such as hydrogen peroxide, urea and glucose. The effective driving force of the biocatalytic nanomotors was measured to be 64 ± 16 fN with high-resolution optical tweezers, which is expected to be further used in directional cargo delivery. In another work, Van Hest and coworkers reported an out-of-equilibrium enzymatic reaction network to propel the micromotors (Nijemeisland *et al*. 2016). The four cycles of enzymatic reactions enabled the micromotors with tunable and sustained movement using natural glucose and lactate in the body fluids as the fuel.

Apart from direct bubble propulsion, enzyme-powered micromotors can also be activated by bubble-induced buoyancy force. Mann and coworkers reported a catalase-containing organoclay/DNA semipermeable microcapsules with multi-function including flotation of macroscopic objects, biocatalyst delivery and self-sorting of mixed protocell communities (Kumar *et al*. 2018). The microcapsules were fabricated by extruding the polyanionic dsDNA (from salmon testes) and catalase with a syringe and dispersing in the exfoliated aminopropyl-functionalized magnesium phyllosilicate (AMP) clay sheets, which gave rise to the electrostatically induced self-assembly of the microcapsule formation. Co-encapsulation of glucose oxidase to the original microcapsules was exploited to induce sustained oscillatory vertical motion, which decreased the buoyant force by consuming the oxygen microbubbles. The proposed microcapsules exhibited great motility and flexibility for sufficient cargo delivery, which can be further manipulated with remote magnetic guidance.

Apart from the commonly used bubble propulsion to drive enzyme-powered micromotors, a non-bubble propelled protein microtube motor was applied by Komatsu and coworkers in the sodium phosphate buffered solution using urea as the fuel (Fig. 2C) (Sugai *et al*. 2019). The microtubes were fabricated by template synthesis using polycarbonate (PC) membrane with poly(L-arginine) (PLA), human serum albumin and avidin, followed by biotinylated urease coupling onto the internal wall with avidin–biotin interaction. With the diffusion of NH_3_ and CO_2_ produced from urea, the microtubes exhibited straight motion with non-bubble propulsion and repetitive lateral self-rotation.

The biocompatible MNMs manufacturing is also applicable using printable materials and printing technology (Fig. 2D) (Gregory *et al*. 2016). Ebbens and coworkers reported a bubble-propulsive micro-rockets fabricated by inkjet printing by alternating printing of a silk/catalase/polyethylene glycol (PEG) ink and a methanol ink. The proposed reactive inkjet printing was used to alter the distribution of catalase to form asymmetrically propelled micromotors which moved faster in aqueous environments in the presence of hydrogen peroxide fuel. This simple and facile printing technology is expected to open up new potentials in lab-scale lithographic fabrication processes for miniaturized devices.

### Water treatment

Taking advantage of the mobility, reusability and ease of collection, self-propelled MNMs are widely investigated in water remediation for heavy metal capture (Jurado-Sánchez and Wang 2018). However, most of the MNMs proposed for water treatment employ metallic catalysts for propulsion, such as cobalt and platinum (Parmar *et al*. 2018; Ying *et al*. 2019), which poses the risk of introducing additional pollutants that is harmful to the environment. Biofriendly materials operating on enzyme catalysis can circumvent this issue by eliminating the need for metallic catalysts and simultaneously exploiting molecules that are innate in the environment as chemical fuels to drive their motion (Wong *et al*. 2019). Up until now, only a few reports have employed biocatalytic MNM systems in environmental remediation.

**Figure 3:**
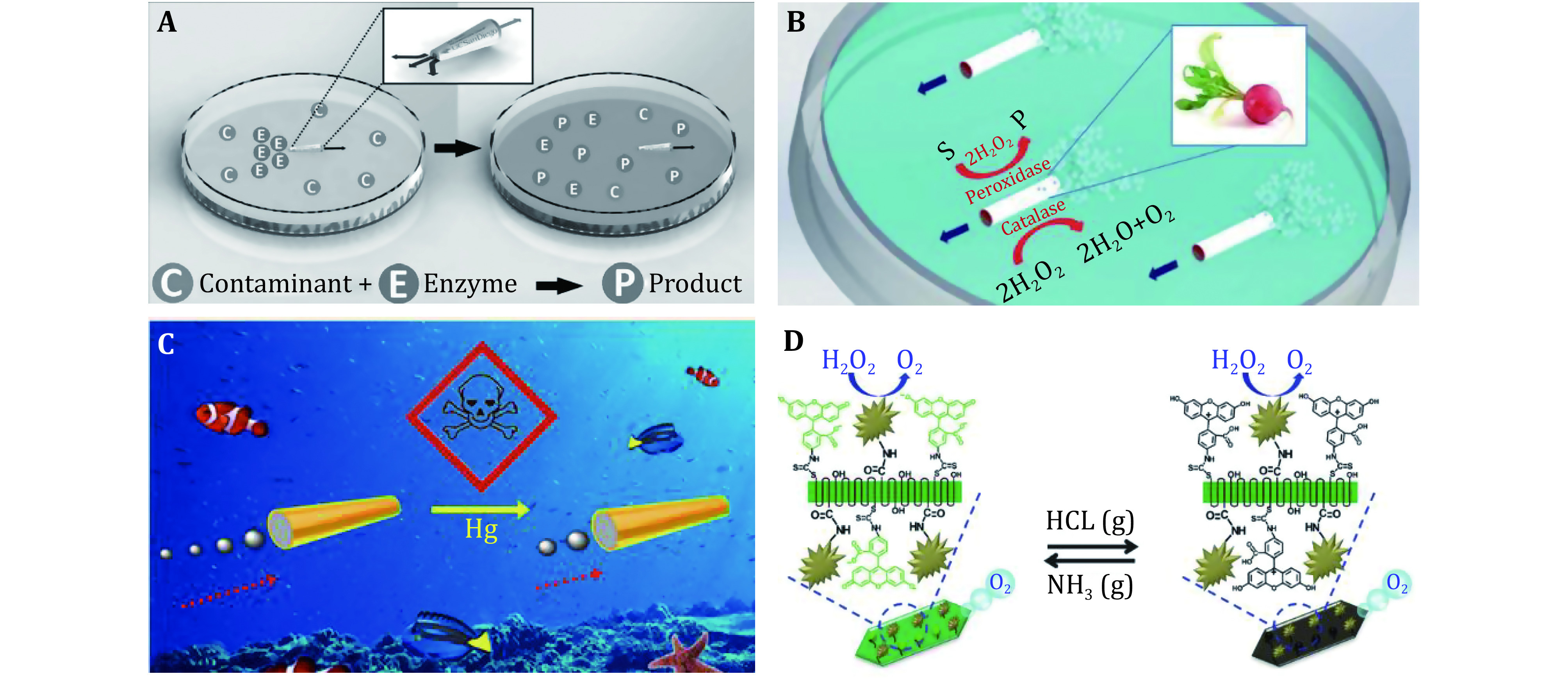
Micromotor systems working in the natural environment. **A** Schematic illustration of the concept of motor-based biocatalytic pollutant remediation involving gradual release and mixing of an enzyme. Reproduced with permission of WILEY-VCH (Orozco *et al*. 2014). **B** Schematic illustration of the dual-function plant (radish) motors. Reproduced with permission of the Royal Society of Chemistry (Sattayasamitsathit *et al*. 2014). **C** Schematic illustration of the pollutant effect on the microfish locomotion speed. Reproduced with permission of American Chemical Society (Orozco *et al*. 2013). **D** Schematic representation of self-propelled all-polymer micromotor and the gas sensing behavior. Reproduced with permission of the Royal Society of Chemistry (Liu *et al*. 2016)

The work reported by Wang and coworkers first demonstrated a tubular motor made from commercial pipette tips filled with laccase solution and sodium dodecyl sulfate (SDS) solution (Fig. 3A) (Orozco *et al*. 2014). This self-propelled microsystem presented a new biocatalytic decontamination strategy by randomly releasing environmental remediation agent while moving. Propelled by the Marangoni effect, the tubular micromotors could navigate in a contaminated solution with effective fluid convection and gradual enzyme release. The well-dispersed enzymatic remediation agent laccase showed great removal efficiency of phenolic pollutant and the motor system was further applied for heavy metal cleanup with substitutive complexing agent EDTA.

In another work from Wang’s group, a fully natural biomotor was made of plant tissues cherry-belle radish (*Raphanus sativus*), which was rich with catalase and peroxidase enzymes (Sattayasamitsathit *et al*. 2014) (Fig. 3B). Hydrogen peroxide was applied to power the motion of the motors catalyzed by catalase, as well as assisting the transformation of toxic phenolic pollutants as a co-substrate. The motion-induced convection resulted in faster environmental remediation. With additional attachment of catalase to the biomotor, the required peroxide fuel concentration could be dramatically reduced.

### Chemical sensing

Apart from wastewater treatment, the activated motion behavior of biocatalytic MNMs could be used for detecting the presence of aquatic pollutants and assessing the water quality (Jurado-Sánchez and Wang 2018). The great flexibility and mobility of MNMs also endowed the system with great potential for probing solutes in the solutions. Most of the existing MNMs reported were propelled by metallic catalysis including platinum, gold, and magnesium (Jurado-Sánchez and Escarpa 2017; Pacheco *et al*. 2019). However, biocatalytic MNMs have also been demonstrated as a suitable platform for chemical sensing in aqueous environment.

Wang and coworkers reported a tubular biocompatible microfish using poly(3,4-ethylenedioxythiophene) (PEDOT) microtubes as the backbone and anchoring the catalase enzyme on a mixed self-assembled binary monolayer coupled with EDC/NHS for oxygen bubble propulsion (Fig. 3C) (Orozco *et al*. 2013). The locomotion and the swimmer survival time were in accordance with the quantity of the contaminants, in response to the impairment of the enzymatic activity. This artificial microfish is expected to offer a real-time sensing capability for water quality by reflecting the presence of heavy metal, pesticide and herbicide.

Li and the coworkers described an enzyme-powered biodegradable micromotor system for gas sensing applications (Liu *et al*. 2016) (Fig. 3D). The micromotor was fabricated with polycaprolactone as the backbone. Catalase and fluorescein isothiocyanate (FITC) were immobilized on the motor surface as the biocatalytic engine and signal indicator, respectively. Due to the pH-responsive nature of FITC as a result of fluorescence intensity fluctuation, the micromotors were applied for acidic or basic gas molecule sensing including hydrogen chloride and ammonia.

### Nanomedicine

A significant number of studies have been reported on the enzyme-powered MNMs for nanomedicine applications (Wang *et al*. 2019b). Amongst them, catalase catalyzed bubble propulsion using H_2_O_2_ as the fuel is one of the most practicable mechanisms (Gao *et al*. 2019; Guo *et al*. 2019; Wu *et al*. 2015). Although the excessive amount of the H_2_O_2_ fuel is not biofriendly, the distinct elevated concentration of H_2_O_2_ fuel in tumor and inflamed tissues could sufficiently enhance MNM movement, promoting drug delivery and motion-activated targeting mechanism (Safdar *et al*. 2018).

**Figure 4:**
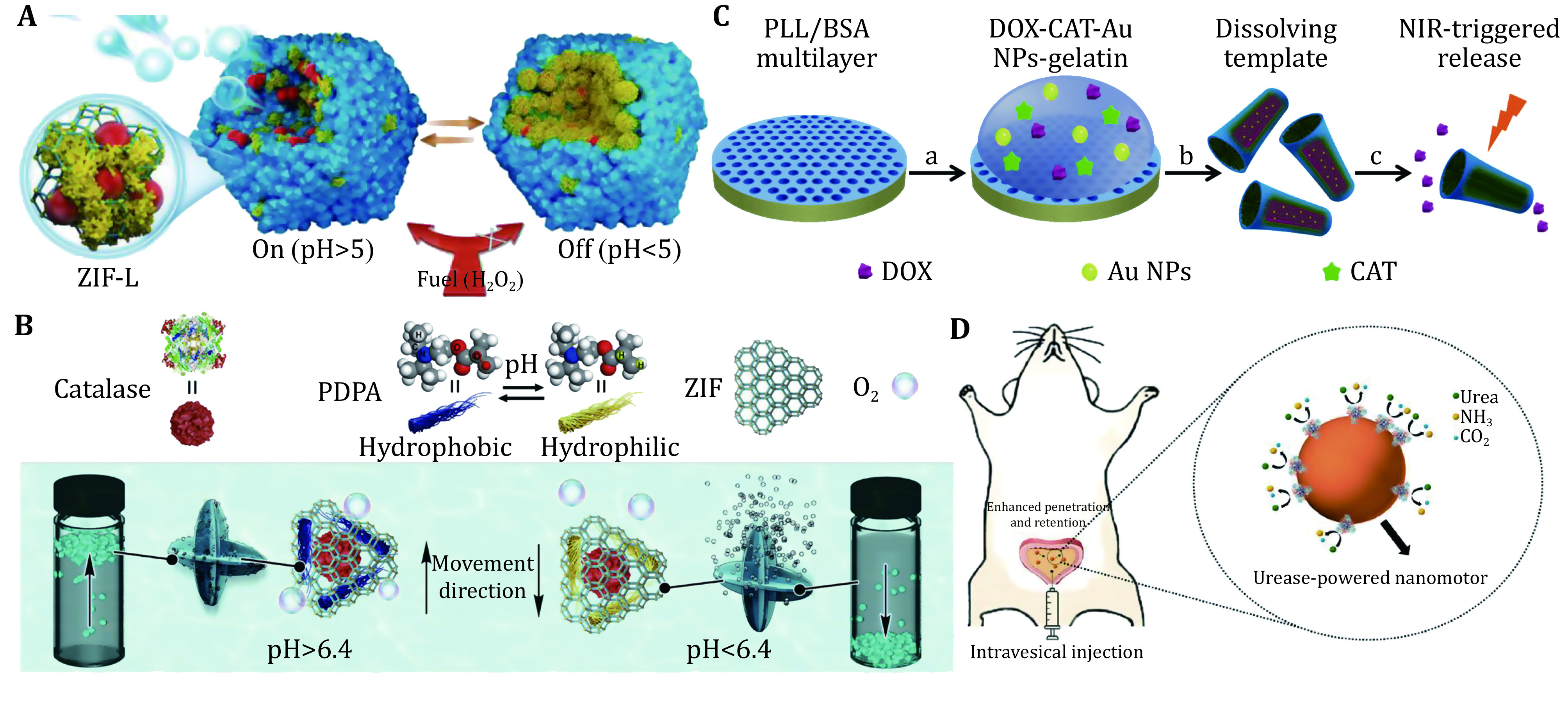
Micromotor systems for drug delivery. **A** MOFs-based micromotors with reversible pH-speed regulation. Reproduced with permission of WILEY-VCH (Gao *et al*. 2019). **B** Vertical directionally propelled micromotors with pH-controlled hydrophilicity/hydrophobicity switch. Reproduced with permission of Elsevier (Guo *et al*. 2019). **C** PLL/BSA-Based rockets for drug transportation and light-triggered release. Reproduced with permission of American Chemical Society (Wu *et al*. 2015). **D** Schematic illustration of intravesical delivery using urease-powered nanomotors. Reproduced with permission of American Chemical Society (Choi *et al*. 2020)

Our group developed a MOF-based biocatalytic nanomotor with pH-controlled reversible-speed regulation (Fig. 4A) (Gao *et al*. 2019). Catalase and succinylated β‐lactoglobulin were encapsulated in nanoporous MOF particles as the engine and gear, respectively. At neutral pH, the H_2_O_2_ fuel could access the catalase through the MOF porous network and produce adequate oxygen bubbles, which could sufficiently propel the MOF nanomotors with thrust. While at mild acidic pH, the β‐lactoglobulin underwnet a gelation process, which could block off the MOF pores and hinder the H_2_O_2_ fuel from getting access to catalase, causing diminished nanomotor motion. Results indicated that the cytotoxicity of the doxorubicin-loaded micromotor was originated from two steps. Firstly, drugs were partially released from the motors extracellularly when the accelerated nanomotor motion was triggered in the elevated H_2_O_2_ local environment. Secondly, upon cellular uptake, the MOF-based motor was degraded in the cellular acidic compartments which further released the drugs inside of cell. In another work, we designed a biocatalytic self-propelled submarine-like micromotor with buoyancy controlled directional vertical motion for pH-controlled drug delivery (Fig. 4B) (Guo *et al*. 2019). The micromotor was constructed with zeolitic imidazolate framework-L (ZIF-L), simultaneously encapsulating bioactive enzyme catalase as the engine and a pH-sensitive polymer poly(2-diisopropylamino)ethyl methacrylate (PDPA) as the switch. In neutral pH environment, the micromotors could retain the oxygen bubbles catalyzed from catalase at the motor–water interface to produce buoyancy force to efficiently drive the micromotor upward. When it was switched to slightly acidic environments, the PDPA underwent hydrophobic/hydrophilic structural transformation and released the oxygen bubbles, resulting in micromotor downward motion. Consequently the proposed micromotors were capable of targeted delivery of anti-cancer drug 5-FU vertically to the cancer cells at different spatial locations controlled by versatile pH switches.

Considering the drawbacks of toxicity of H_2_O_2_ as chemical fuels, some other studies adapted alternative ways to enhance the motors’ biocompatibility by exploiting more biofriendly biomolecules such as glucoses (Schattling *et al*. 2015) and urea (Dey *et al*. 2015; Hortelão *et al*. 2018; Ma *et al*. 2015) as the fuel. Städler and coworkers developed a glucose-propelled Janus particle system with an in-built enzyme cascade reaction (Schattling *et al*. 2015). Glucose oxidase (GOx) and catalase were co-immobilized in the particles and the biocompatible fuel glucose was decomposed by GOx to produce H_2_O_2_, followed by the catalytic reaction of catalase to form oxygen bubbles. The cascade reaction resulted in lower power output to the mobile system compared to that of direct usage of H_2_O_2_ fuel and inorganic-based catalyst, but the intermediate product H_2_O_2_ was kept at a safer level for the benefit of the systematic biocompatibility. Considering the biocompatibility of both the motor and the chemical fuel, this system is believed to be suitable for *in vivo* applications, particularly as self-propelled drug delivery. In a following work, the authors expanded the biocompatible Janus micromotor system to a double-fueled system with glucose and peptides, which enhanced the power of engines without compromising their biocompatibility (Schattling *et al*. 2017).

The precise controlling of the drug release is also one of the main MNMs research priorities in nanomedicine. To this end, Wu and coworkers reported a poly-L-lysine/bovine serum albumin (PLL/BSA) multilayer rocket consisted of heat-sensitive gelatin hydrogel, gold nanoparticles, doxorubicin and catalase (Fig. 4C) (Wu *et al*. 2015). The drugs were delivered to the cancer cells through the biocatalytic bubble propulsion with additional precision by magnetic guidance. When they arrived at the cancer cells, exposing the motors to NIR irradiation could produce heat as a result from the photothermal effects of gold nanoparticles and dissolve gelatin to release doxorubicin to the surrounding cancer cells. With great flexibility, biodegradability and multifunctionality, this micromotor system showed a promising future for *in vivo* drug delivery.

So far, most of the works involving MNMs for drug delivery were based on *in vitro* two-dimensional cell culture, while the micro-environments for practical *in vivo* drug delivery are much more complex. Sanchez and coworkers took a step forward and built a urease-powered nanomotors with anti-FGFR_3_ antibody on the outer surface (Hortelão *et al*. 2019). The micromotors were propelled with urea and could specifically target the 3D spheroids bladder cancer cells, which was believed to be a closer model to the real tumor *in vivo*. The proposed nanomotor carrier showed a significantly higher internalization efficiency into the spheroids compared to that of passive particles and presented enhanced cancer therapy effect.

Very recently, Hahn and the coworkers reported a urease-powered nanomotor for intravesical therapy of bladder diseases (Choi *et al*. 2020) (Fig. 4D). The nanomotors were constructed with polydopamine (PDA) nanocapsules on sacrificial silica nanoparticles, which were then etched away with hydrofluoric acid (HF). Urease was attached to the PDA surface through Schiff base reaction between amine groups on urease and catechol groups on PDA. Propelled by the biocatalytic conversion of urea, the nanomotors could penetrate into the bladder tissue and prolong the retention time after repeated urination. This nanomotor system could be used as sustained release drug carriers for bladder diseases.

Wang and coworkers reported an enzyme-powered cell robots with natural platelet cells (Tang *et al*. 2020). The biocatalytic enzyme urease was grafted asymmetrically on the surface of the bare cells to enable the chemophoretic motion. The surface functionalized platelet cells exhibited intrinsic biofunctionalities of cancer cell targeting, which is sufficient for targeted drug delivery. The hybrid system combined the merits from both biocompatible enzymatic rection-induced motion and the intrinsic capacity of natural cells, offering inspiring avenues for biocompatible MNMs system development.

Despite the rapid progress, enzyme-powered MNM for nanomedicine is still in the early proof-of-concept phase. Very limited researches have assessed enzyme-powered MNM systems *in vivo* although other kinds of MNMs have been reported for *in vivo* drug delivery, such as metal catalyst-based MNMs (de Ávila *et al*. 2017; Esteban-Fernández de Ávila *et al*. 2018a; Li *et al*. 2017). It may be attributed to the instability of enzymes when they are delivered inside the body, leading to hydrolyzation and degradation by the living organism. In addition, less biofriendly fuel such as H_2_O_2_, needs to be kept at lower level or replaced by other more biofriendly fuels. Nevertheless, with the research of new nanomaterials that can protect enzymes even inside the body (Guo *et al*. 2020; Liang and Liang 2020), enzyme-powered MNMs are expected to open a new era in nanomedicine.

### Imaging and biosensing

**Figure 5:**
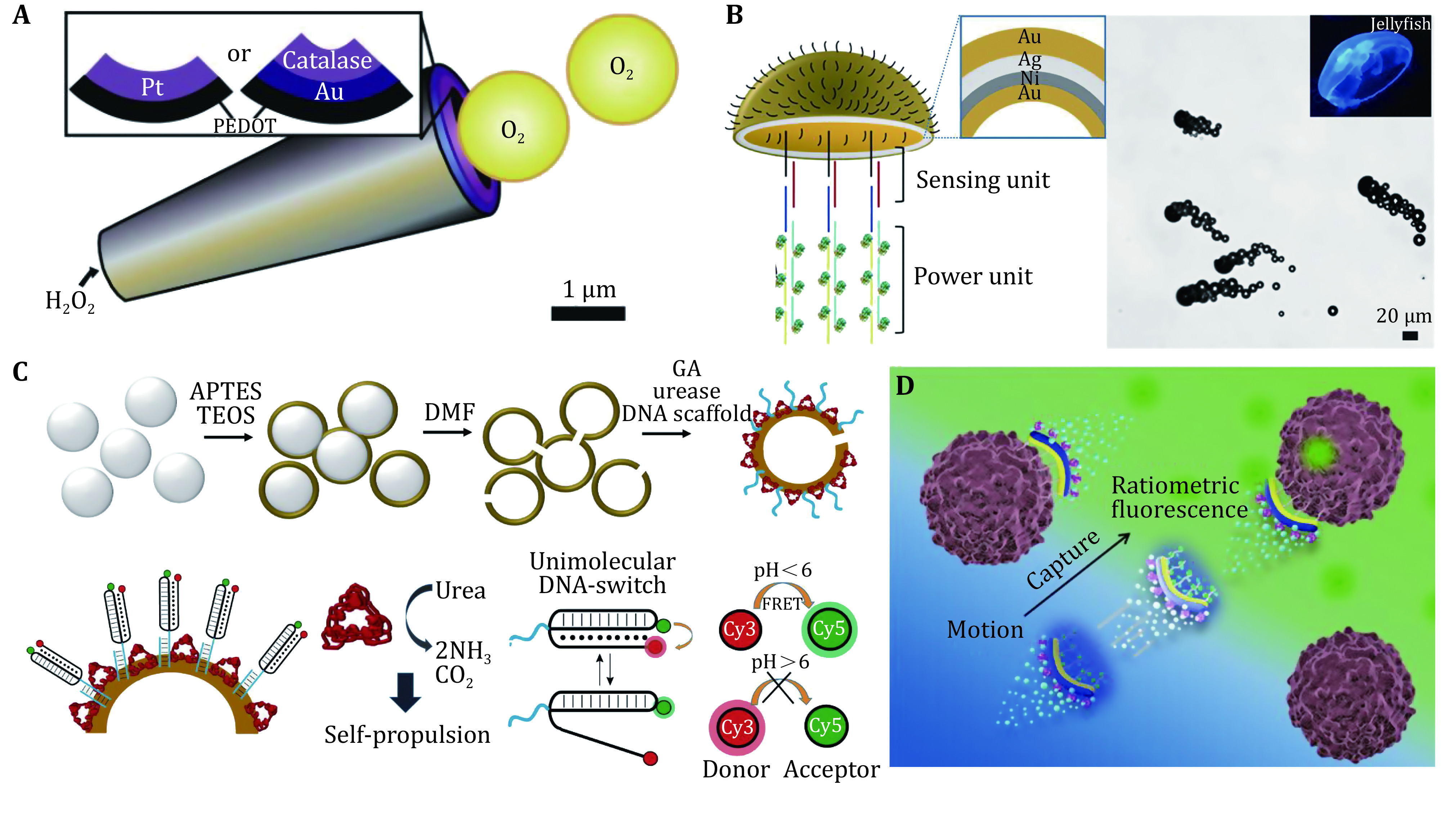
Micromotor systems for biosensing. **A** Schematic illustration of ultrasound-based visualization of oxygen microbubbles formed by micromotor converters. Reproduced with permission of Elsevier (Olson *et al*. 2013). **B** Schematic illustration of micromotors used for specific DNA sensing. Reproduced with permission of American Chemical Society (Zhang *et al*. 2019). **C** Schematic representation of the micromotors fabrication, where a silicon dioxide layer is grown onto a commercial polystyrene template. Reproduced with permission of American Chemical Society (Patino *et al*. 2019). **D** Schematic illustration of ratiometric fluorescence response of Janus micromotors after capture of tumor cells. Reproduced with permission of Elsevier (Zhao *et al*. 2020)

Bioimaging techniques are widely accepted as the core of prompting diagnosis, which played an important role in assessing the progress of disease and precise surgery preparation (Sun *et al*. 2017; Wang *et al*. 2018). Biomacromolecules and micro-environments of tumor cells are often recognized as the biomarkers of cancer (Felder *et al*. 2014). In recent years, tremendous attention has been paid to the development of bioimaging enhancement with MNMs (Peng *et al*. 2017). Mattery and coworkers reported nanomotor assisted ultrasound imaging in the abscess in rats (Fig. 5A) (Olson *et al*. 2013). Poly(sodium styrene sulfonate) (PSS) was used as the motor matrix with negative-charged surface, which could be further functionalized for cell targeting, and catalase was immobilized as the biocatalytic engine with local H_2_O_2_. The locally produced microbubbles were considered as specific signals for activated neutrophils in ultrasound imaging with high sensitivity. Based on this mechanism, this micromotor system was able to distinguish activated neutrophils from native ones due to the lack of H_2_O_2_ in native neutrophils. According to the bioimaging results of injected micromotors in the abscess of rats, the groups with activated neutrophils showed an enhanced ultrasound signal compared to the conventional microscopy techniques.

Apart from bioimaging, MNMs assisted biosensing was also well discussed and accepted in disease diagnosis (Jurado-Sánchez and Escarpa 2017; Kong *et al*. 2018). Some efforts have been made to fulfill the need of early-stage tumor detection with the MNMs (Gao *et al*. 2018; Wang *et al*. 2019a). Ju and coworkers recently developed a jellyfish-like micromotor with DNA detecting assembly and biocompatible enzyme catalase, which was decorated on the concave surface of the motor (Fig. 5B) (Zhang *et al*. 2019). The motor exhibited quick moving behavior in bio-media from the catalytic reaction of catalase in the presence of H_2_O_2_. When the specific DNA sequences were detected and matched by the DNA self-assembly, the micromotor disassembled and resulted in the detachment of the catalase. Therefore, the proposed system was applicable in DNA sensing with the speed of motion corresponding to the presence of the target DNA. This micromotor provided a facile method to detect the biomacromolecules with a good sensitivity and reproducibility.

Sánchez and coworkers reported a urease-powered micromotor with synthetic DNA nano-switch which acted as a photostable FRET-based probe (fluorescence resonance energy transfer) (Fig. 5C) (Patino *et al*. 2019). This sensor could provide real-time monitoring of micro-environment pH changes through FRET/Cy3 (cyanine-3 fluorophore) ratio fluctuation in a few seconds. The versatile platform was proved to be applicable in intracellular pH which is suitable for sensing the tumor micro-environment.

In another work, Zhao and coworkers reported Janus micromotors for circulating tumor cells detection with motion-capture-ratiometric fluorescence changes (Fig. 5D) (Zhao *et al*. 2020). The micromotor system was propelled by decomposing hydrogen peroxide with catalase, which was grafted on one side of the Janus rods, and TLS11a aptamers were decorated on the other side of Janus rods for cell targeting. To achieve fluorescence indication, thymine and guanine were conjugated on tetraphenylethene (TPE) and fluorescein isothiocyanate (FITC) followed by grafting onto aptamers via basepair interactions. Due to the aggregation-induced emission (AIE) of TPE and aggregation-caused quenching of FITC, the competitive binding of tumor cells with aptamers resulted in the fluorescence changes from blue to green. This motor system demonstrates the capabilities of motion-capture ratiometric fluorescence detection of tumor cells with high selectivity, rapid recognition, and low detection limit.

## CONCLUSION AND PERSPECTIVES

In this review, we capture the recent research efforts in biocompatible MNMs that operate on enzyme-powered catalysis with different constructions and applications ranging from biomedicine to the environment. The rapid explosion of nanomaterial research in the past two decades has enabled diverse propulsion mechanisms and possible applications. At present, the full promise of MNMs constructs to real-world applications has still not been met as the field has only begun to mature recently. One of the major challenges remained for real-world application is the compatibility of MNMs with the biological system and the natural environment. There are still works to be performed on designing new techniques and strategies for integrating biocompatible MNMs with diverse applications. Another important consideration in engineering these MNMs is the endogenous available energy source or, at least, biofriendly fuels available for their motion. The excessive amount of fuels required for MNM motion could be harmful to the biological system and natural environment. Therefore, it is important to develop more energy efficient motors with less fuel demand. The current biocompatible MNMs generally revolve around enzyme biocatalysis, particularly catalase for decomposing hydrogen peroxide. In contrast, biological system utilizes thousands of different enzymes to catalyze diverse biochemical reactions. This represents new opportunity for MNMs by exploiting new biocatalytic pathways. In addition, the optimization of MNMs properties — such as size, morphology, geometry, and catalyst distribution — has shown great influence on the motion performances, which should play an important role in guiding or enhancing MNMs development. It is anticipated that advancements in these areas will lead to an improved ability in designing and optimizing biocompatible MNMs that can be ultimately employed for many applications.

## Conflict of interest

Ziyi Guo, Jian Liu, Da-Wei Wang, Jiangtao Xu and Kang Liang declare that they have no conflict of interest.
